# Effect of Electrolyte, Applied Voltage, and DNA Concentration on the Translocation Dynamics of a Single-Stranded DNA through a Solid-State Nanopore

**DOI:** 10.1021/acsomega.6c03954

**Published:** 2026-08-13

**Authors:** Agnes Zerolová, Shubham Mondal, Petteri Piskunen, Mauri A. Kostiainen, Veikko Linko

**Affiliations:** † Department of Bioproducts and Biosystems, Aalto University, 00076 Aalto, Finland; ‡ Institute of Technology, University of Tartu, 50411 Tartu, Estonia

## Abstract

Solid-state nanopores enable label-free detection of biomolecules by monitoring ionic current modulations during molecular translocation. However, reliable measurements are often limited by rapid, poorly controlled translocation dynamics producing short, low-resolution signals. Applied voltage, buffer composition, and analyte concentration influence molecular transport, yet their combined effect in ionic environments, including Tris-based buffers, remains insufficiently understood. Here, we investigated the effects of applied voltage, DNA concentration, and buffer composition on translocation kinetics of a circular 7560-nucleotide single-stranded DNA and the current blockage characteristics. We compared two electrolyte environments: lithium chloride in HEPES (LiCl/HEPES) vs Tris-borate-EDTA (TBE) supplemented with K^+^ and Mg^2+^ cations. The two electrolyte environments differed in ion composition and ionic strength, leading to distinct translocation behaviors. With increasing absolute applied voltage, dwell times decreased and current blockages increased for all DNA concentrations. LiCl/HEPES buffer resulted in slower translocation with broad dwell time distributions, whereas TBE with K^+^ and Mg^2+^ cations produced faster events with narrower dwell time populations and lower current blockage amplitudes. We conclude that the electrolyte environment is a key factor for optimizing solid-state nanopore performance and improving the reliability of single-molecule sensing.

## Graphical abstract

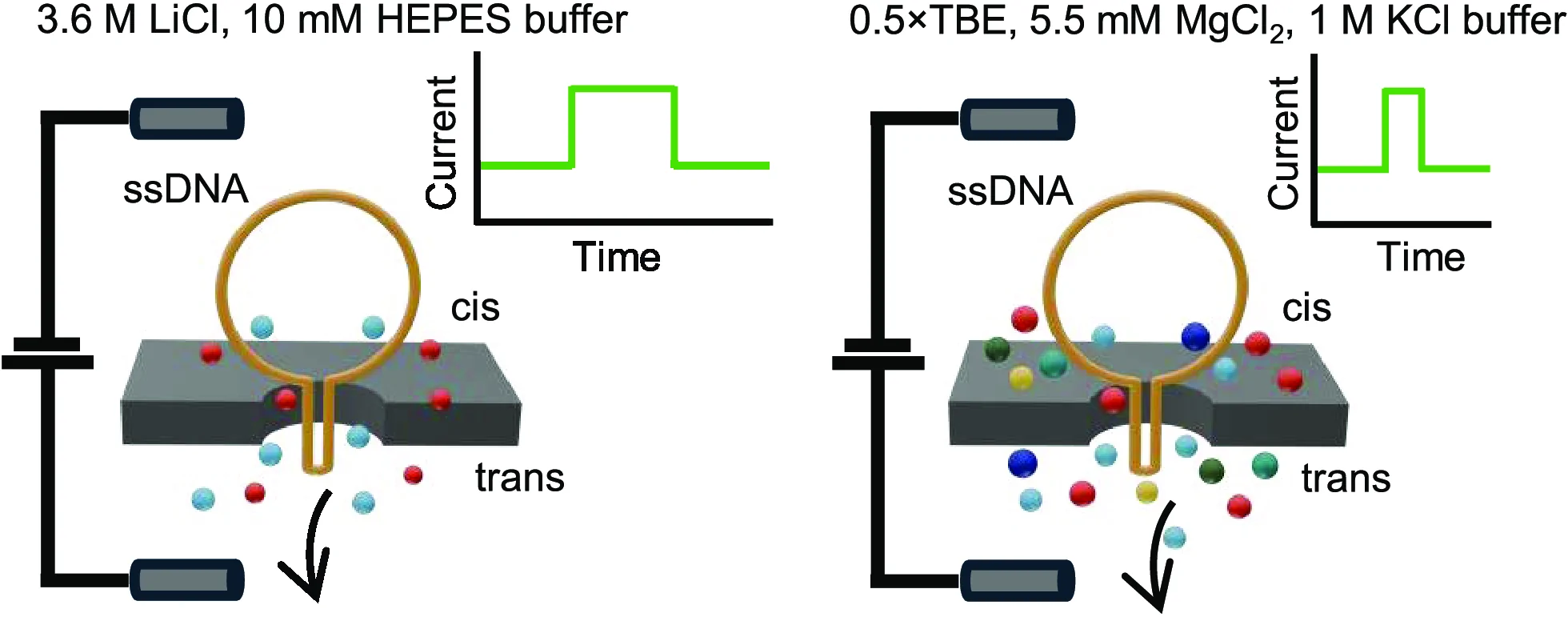

## 1 Introduction

Solid-state nanopore (SSNP) technology has become a promising platform for single-molecule biosensing, finding wide application in various fields of research.^1,2^ In recent years, significant progress has been made in the field of solid-state nanopores, particularly in DNA, RNA, and protein sequencing applications and in single-molecule analysis in general. Recent studies highlight the rapid development of methods enabling higher signal resolution, better control of translocation, and a broader range of analyzed biomolecules.^3–6^ The main challenges that persist in the field include controlling the translocation rate, achieving sufficient signal resolution, and minimizing nonspecific adsorption of analytes on the pore surface.^3^

Although DNA sequencing remains SSNP’s primary use, the technology has also been extended to other areas, including protein analysis and virus detection.^1,2^ In this context, SSNPs enable the label-free detection and analysis of biomolecules with single-molecule resolution.^7–10^ The application of nanopores is particularly significant for the next-generation DNA sequencing, as this method offers label-free and PCR-free single-molecule analysis.^1,2,11,12^ It enables longer read lengths, high throughput and cost-effectiveness.^2,11,12^ In addition to DNA sequencing, SSNP technology has proven to be crucial for the detection and analysis of proteins and protein complexes, including the identification of conformational changes.^1,13^ Although progress in protein sequencing using nanopores has been slower than for DNA, SSNPs show promise for both protein and RNA sequencing.^2,12,13^

SSNP analysis relies on ion current measurements.^7,14,15^ Upon the application of voltage, the passage of the analyte through the nanopore gives transient current blockages, which provide information on the molecular size, charge, and conformation.^16–20^ The quantitative interpretation of these current blockages has been the subject of considerable methodological development, resulting in the availability of comprehensive approaches for modeling the magnitude and shape of blockage events as a function of pore geometry and analyte properties.^21,22^ SSNP fabrication may be accomplished using a variety of methods that allow for fine control over the pore size and shape, such as focused ion beam (FIB) drilling and transmission electron microscopy (TEM), which enable subnanometer accuracy.^7,14,23^ Controlled dielectric breakdown (CDB) is a relatively new technique that has been considered as a simple, inexpensive, and scalable alternative to traditional beam-based fabrication.^7,14,15,24^ A conductive channel is formed in an electrolyte by applying a high electric field across a thin dielectric membrane, typically silicon nitride (SiN). Real-time monitoring of the ionic current allows for precise control over pore development and therefore the desired pore dimensions.^7,23,25^ Silicon nitride (SiN) nanopores are of particular interest due to chemical tunability, thermal stability, and mechanical strength.^23,25^

Notwithstanding the advantages, two persistent challenges limit the development of solid-state nanopores: poor operational stability and lack of molecular selectivity. For electrolyte buffers, nonspecific adsorption, clogging, and degradation during long-term operation may affect reproducibility.^7,24,26^ SSNP properties are typically characterized with translocation experiments where charged molecules pass electrophoretically through the pore and produce transient current blockages dependent on molecular and pore parameters.^7,14,18,19,27^ Beyond the inherent material and geometric properties of the nanopore, its behavior can be further modified by functionalization to enhance selectivity and control transport, for example, by chemical modification of the pore surface. An alternative and rather intriguing strategy is to functionalize the pore by integrating fully programmable DNA nanostructures, such as DNA origami, into pores, which would enable precise modifications of the local environment of the nanopore and specific interactions with the analyte.^28–32^ Despite these advances, one of the main limitations of SSNP technology may still remain; the limited control over translocation dynamics.^7,12,33,34^ Molecules typically pass through the nanopore on microsecond time scales, resulting in short, low-resolution signals that hinder accurate identification and reduce analytical sensitivity.^7,12,33,34^ Variations in dwell time and current blockage amplitude, resulting from differences in molecular conformation and their interactions with the nanopore, complicate signal processing and reduce the reproducibility of measurements. Thus, controlling the velocity and consistency of translocation is essential for improving signal resolution and enabling reliable single-molecule analysis, and therefore it represents one of the key challenges in current solid-state nanopore research.^7,12,33–37^

Studies focusing on DNA translocation in electrolytes containing monovalent cations (K^+^, Na^+^, Li^+^) through SSNPs have shown that these cations slow down single-stranded DNA (ssDNA) and double-stranded DNA (dsDNA) translocation, with the effect increasing in order from K^+^, Na^+^ to Li^+^.^38,39^ Skinner et al. directly compared the translocation of ssDNA and double-stranded RNA (dsRNA) in a 10 nm SiN nanopore and demonstrated that interactions between the molecule and the nanopore surface can lead to the formation of subpopulation of events with unusually long dwell times and weak dependence on the applied voltage.^40^ It has also been demonstrated that a long ssDNA with a random sequence can adopt compact, partially folded conformations in solution through intramolecular base pairing, forming blob-like structures that may need to unravel before passing through the pore, leading to a translocation mechanism and voltage dependence qualitatively distinct from linear or double-stranded DNA.^41,42^ Recent studies further show that translocation dynamics are determined not only by the applied voltage but also by stochastic rate fluctuations and interactions between the molecule and the nanopore, which can lead to significant variability in experimentally observed signals.^43^ Uplinger et al. and Kowalczyk et al. demonstrated that Na^+^ and Li^+^ bind more strongly to DNA than K^+^, and that different binding strengths affect the effective dragging force of DNA, and thus the velocity of translocation.^38,39^ At high concentrations of LiCl, it has been demonstrated that DNA translocation proceeds in an entropy-limited regime, distinct from the drift-dominated regime observed at lower salt concentrations, such as 1 M KCl, which may further contribute to the pronounced differences in dwell time observed between LiCl- and KCl-based electrolytes.^30^

It has been demonstrated that SiN nanopores fabricated with CDB method can exhibit enhanced interactions between DNA and the nanopore surface, and the character of these interactions may depend on the electrolyte composition and the presence of polyvalent ions in the solution, a factor that must be taken into account when interpreting translocation data.^44–47^ Moreover, as divalent Mg^2+^ binds more tightly to DNA than K^+^, addition of low concentrations of Mg^2+^ to KCl solution efficiently neutralizes the charge of DNA and reduces its mobility.^38^ In mixed salt solutions containing both Na^+^ and Mg^2+^, the conformation of ssDNA is influenced by ion-specific charge shielding resulting from the simultaneous presence of monovalent and divalent cations. While Mg^2+^ promotes more compact ssDNA conformations through stronger electrostatic interactions with the phosphate backbone, Na^+^ modulates the local electrostatic environment in a weaker manner. The resulting effect on nanopore translocation dynamics depends strongly on the relative concentrations of both ion species.^48^ DNA translocation dynamics through solid-state nanopores are impacted not only by changes in DNA’s effective charge due to the counterion screening, but also by DNA conformation and length, and naturally, also by the nanopore size.^38,39^ However, the available literature describing translocation in common single-buffer systems used in molecular biology is more limited. The reported translocation times for single-stranded nucleic acids can vary significantly depending on experimental conditions, electrolyte composition, and the extent of interactions between the molecule and the nanopore; translocation times ranging from tens of microseconds to milliseconds have been reported in the literature.^49,50^ The effect of Tris-based systems on SSNP translocation has not been extensively studied in comparison with monovalent cations. Nevertheless, DNA mobility in Tris-borate-EDTA (TBE) and Tris-acetate-EDTA (TAE) buffers has been studied by gel electrophoresis.^51^ As Tris-based buffer is commonly used in many DNA origami applications (also as a DNA origami folding buffer), understanding translocation behavior of DNA under these conditions might also become useful for integration of DNA origami structures into SSNPs for creating advanced DNA origami nanopore systems.^28,29,52^ The results showed that DNA-borate complexes migrate faster than DNA alone, and with the addition of NaCl, DNA mobility decreased with increasing NaCl concentration.^53^ Recent studies suggest that the influence of the ionic environment cannot be reduced simply to the type of cation, but rather that the buffer composition, the presence of complexing agents, and the interactions of ions with both the DNA and the nanopore surface can significantly affect both the translocation rate and the character of current blockages.^49^ The translocation times reported by Chau et al. and Earle et al. serve as reference values for interpreting the dwell times observed in this study.^49,50^

In this work, we study what the characteristic translocation properties (dwell time and current blockage) of single-stranded and circular DNA are when DNA passes through a solid-state nanopore with a diameter of ∼16 nm produced by the CDB method. These properties are in agreement with the published findings, especially when translocation events are characterized in LiCl. We find that translocation is less clearly detectable in TBE supplemented with K^+^ and Mg^2+^ cations, which we attribute to the combined effect of higher passage speed and reduced signal resolution under these conditions.

## 2 Materials and Methods

Except where noted otherwise, all chemicals were obtained from commercial vendors and used without modifications. For all experimental procedures, deionized water of Milli-Q grade was used.

A circular, single-stranded DNA (circular ssDNA) p7560 (typically used as a DNA origami scaffold strand) was sourced from Tilibit Nanosystems at a concentration of 100 nM. The 50× TBE buffer, consisting of 445 mM tris­(hydroxymethyl)­aminomethane (Tris), 445 mM boric acid, and 10 mM ethylenediaminetetraacetic acid (EDTA), at pH 8.3, was acquired from Thermo Fisher Scientific. Sigma-Aldrich supplied potassium chloride, magnesium chloride and lithium chloride.

### 2.1 Fabrication of Nanopores Using Controlled Dielectric Breakdown

SiN membranes with a thin oxide layer, referred to as low-noise membranes (window thickness: 12 nm ± 2 nm (given by manufacturer), window size: 40 μm × 40 μm, supplier: NORCADA), were utilized as substrates for nanopore fabrication. The chips were mounted in a flow cell situated between the cis and trans reservoirs (Northern Nanopore Instruments). The flow cell contained two reservoirs, which were initially filled with a fabrication buffer consisting of 1 M KCl and 10 mM HEPES solution at pH 8, with a conductivity of 10.6 ± 0.5 S/m (s.d.). Two Ag/AgCl electrodes were immersed in these solutions. Nanopores were fabricated using the Spark-E2 tool and the accompanying software from Northern Nanopore Instruments through controlled dielectric breakdown (CDB) method. The nanopore was first created in the fabrication buffer, and after the initial nanopore was formed, the fabrication buffer was replaced with a conditioning buffer (3.6 M LiCl and 10 mM HEPES solution, also at pH 8 and a conductivity of 16.5 ± 0.5 S/m (s.d.)), which was used to further enlarge the nanopore to the desired size and increase its stability. This process resulted in the creation of a nanopore with a diameter of 15.1 ± 1.0 nm as calculated from an *I*–*V* curve. After fabrication, the nanopores were still allowed to age overnight in conditioning buffer under ambient laboratory conditions to further enhance pore stability. After the stabilization period, the final nanopore size used for translocation measurements was 16.0 ± 1.0 nm based on the calculation from the measured *I*–*V* curve using a conductance model by Kowalczyk et al.^54^ that includes also an access resistance, which is in agreement with the models described by Wanunu et al. and Chinappi et al.^55,56^ All translocation experiments described in this study, in both electrolyte systems, were performed using the same, single nanopore.

### 2.2 DNA Translocation Measurements

Both *cis* and *trans* chambers of the flow cell were filled with either 10 mM HEPES, 3.6 M LiCl solution at pH 8, or 0.5× TBE, 5.5 mM MgCl_2_, and 1 M KCl buffer at pH 8 and a conductivity of 11.86 S/m. Only the solution in the *cis* chamber contained circular ssDNA p7560 at a concentration of 3–5 nM. An Ag/AgCl electrode was immersed in each solution to ensure electrical contact between the chambers. To detect the translocation events of ssDNA, experiments were conducted using a high-bandwidth, low-noise, voltage-clamp amplifier (VC100, Chimera Instruments) at a sampling rate of 4.17 MHz with an analog bandwidth 1 MHz. The ionic current was measured at an applied voltage range of −50 mV to −100 mV, with each measurement lasting 25 s. Between consecutive measurements, the *cis* and *trans* reservoirs were thoroughly rinsed with the working buffer to prevent cross-contamination. For LiCl/HEPES measurements, the acquired data were digitally filtered with a 50 kHz low pass Bessel filter. For Tris-based measurements, a higher digital bandwidth filter of 100 kHz was applied, as the signal was dominated by noise at lower bandwidth settings, consistent with the faster and lower amplitude events observed in this buffer. Even at 100 kHz bandwidth, the noise level in the Tris-based system remained elevated compared to LiCl/HEPES, some detected events near the threshold may therefore represent baseline noise fluctuations rather than genuine translocation events, and events shorter than approximately 10 μs are likely underrepresented. Current blockage (Δ*I*) events were identified and analyzed using Nanolyzer software (Northern Nanopore Instruments) and Poriscope (Version 1.5.0) using an event detection threshold of 500 pA and a minimum event duration criterion of 1 μs.^57^

Histograms (Supporting Information Figures S2 and S3) were plotted using 35 bins. For the dwell time distributions, a normal fit was applied to the log_10_-transformed dwell time values, while the current blockage distribution was approximated by a normal distribution (Figures 2 and 3 and Supporting Information Figures S2 and S3). To improve the quality of the normal fits, events likely associated with the noise contributions rather than genuine translocation events were excluded from the current blockage distributions prior to fitting. For LiCl/HEPES, events below 2.0 nA were excluded from the data in Figures 2e and S2b, events below 3.5 nA were excluded from the data in Figure S2d, and only events in the range of 2.0–2.8 nA were retained for Figure S2f. For the Tris-based buffer, events below 3.5 nA were excluded from the corresponding current blockage distributions (Figures 3d and S3). Statistical analysis for the data in Figures 2b,c and 3b,c was performed using 2-way ANOVA, where the applied factors were voltage and ssDNA concentration, followed by Tukey’s posthoc test for multiple comparisons between groups. Box plots show only statistically significant differences between groups, while insignificant comparisons are not shown. The level of statistical significance is indicated by asterisks (*p ≤ 0.05; **p ≤ 0.01; ***p ≤ 0.001). Distribution plots and histogram fitting were only performed for data sets containing at least 25 detected translocation events to avoid overinterpretation of under sampled measurements in LiCl/HEPES buffer. A random set of 100 events in Tris-based buffer was chosen from each voltage and concentration combination for statistical comparison to guarantee balanced data sets. The random selection was performed only once and was not repeated with different randomly selected subsets. The exact number of analyzed events in each measurement can be found from Table 1.

**Table 1:** Number of Detected Translocation Events at Different Applied Voltages and Circular ssDNA Concentrations for Two Buffer Systems: 3.6 M LiCl with 10 mM HEPES, and 0.5× TBE with 5.5 mM MgCl_2_ and 1 M KCl^a^

Applied voltage (mV)	Concentration (nM)	Number of events (*N*) (3.6 M LiCl, 10 mM HEPES)	Number of events (*N*) (0.5× TBE, 5.5 mM MgCl_2_, 1 M KCl)
–50	3	5	110
–80		29	183
–100		42	328
–50	4	25	146
–80		30	333
–100		64	369
–50	5	27	224
–80		61	416
–100		77	579

a Each value represents the total number of events recorded during a 25 s measurement.

## 3 Results and Discussion

The aim of our study was to investigate how different ionic environments, applied voltages, and concentrations affect the dynamics of translocation and the stability of nanopores. We first discuss the key results of our study, which focuses on the translocation of circular ssDNA p7560 through a SSNP created using the CDB method.

A typical record of circular ssDNA translocation through a SSNP in 3.6 M LiCl/HEPES is shown in Figure 1a. The current trace shows clearly distinguishable transient current peaks corresponding to individual circular ssDNA translocation events. These events are characterized by a stable baseline and a well-defined blockage amplitude, which is characteristic of measurements in lithium chloride buffer. The high amplitude of current blockages, combined with an improved signal-to-noise ratio (SNR), allows clear identification of individual translocation events. To provide a more detailed view, a zoomed-in section of a selected event is shown, which highlights the typical shape of current blockage observed in our experiment, characterized by a sudden blockage onset, a relatively constant current level during the passage of the circular ssDNA through the nanopore, and a subsequent return to the baseline. The dwell time of events is in the order of thousands of microseconds, which corresponds to the slowed translocation of circular ssDNA in LiCl described in previous studies and allows reliable analysis of individual events.^39,58^ In contrast, in the Tris-based system (shown in Figure 1b) the absolute baseline current is lower in magnitude (−6.29 nA vs −8.95 nA in LiCl/HEPES), primarily due to the lower ionic conductivity of the TBE-based buffer compared to LiCl/HEPES.

**Figure 1:**
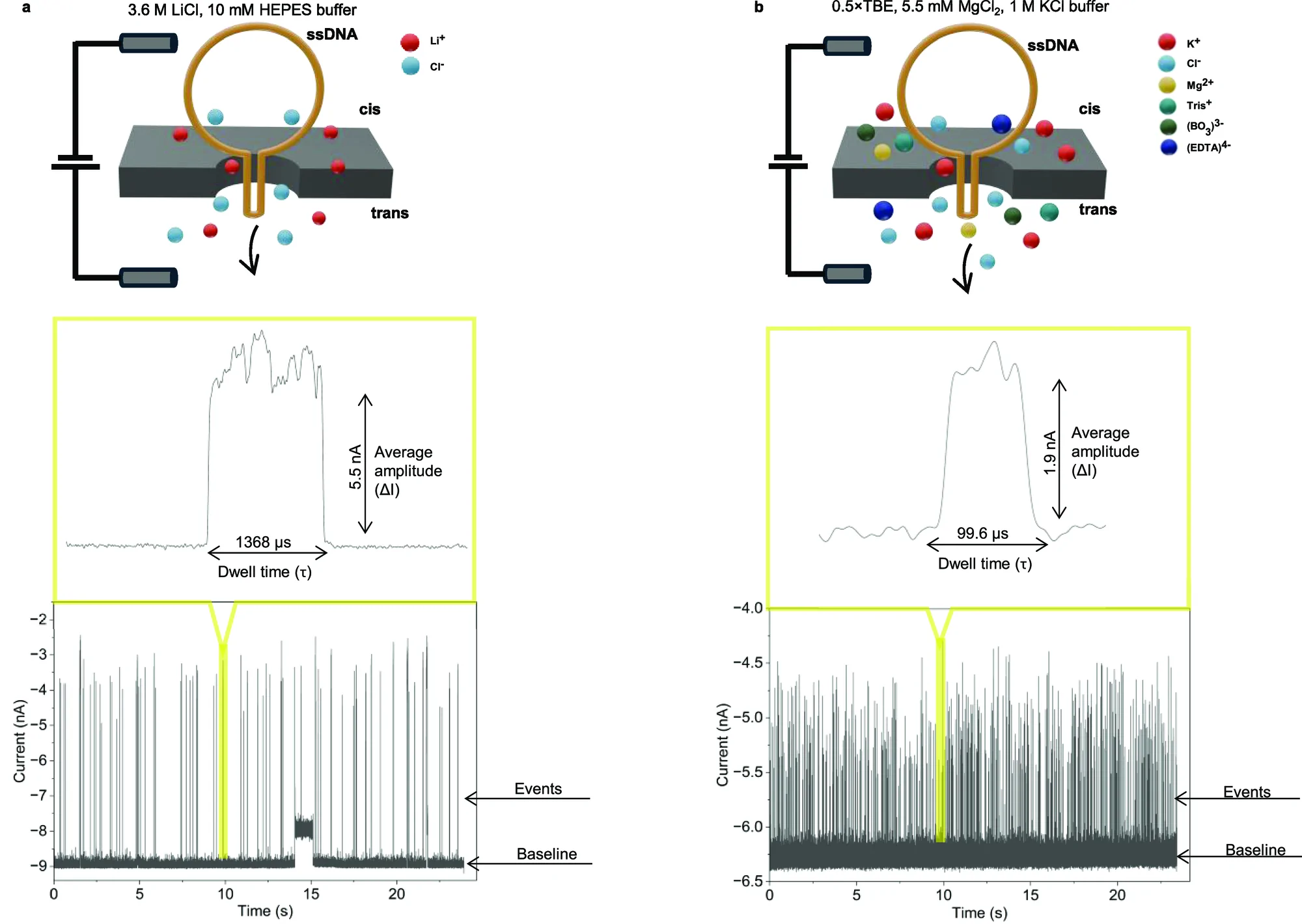
ssDNA translocation through a 16 nm diameter nanopore in two different buffers. All measurements were performed at −100 mV. (a) Schematic presentation of circular ssDNA translocation in 3.6 M LiCl/HEPES buffer, including a representative 25 s recording of ion current and a detail of a single representative translocation event, with the dwell time (1368 μs) and average current blockage (5.5 nA). (b) Schematic presentation of circular ssDNA translocation in 0.5× TBE, 5.5 mM MgCl_2_, and 1 M KCl buffer, including a representative 25 s recording of ion current and a detail of a single representative translocation event, with the dwell time (99.6 μs) and average current blockage (1.9 nA). Under respective acquisition conditions, the baseline RMS noise was 40 pA for the LiCl/HEPES buffer and 50 pA for the Tris-based buffer.

The current blockage amplitude is relatively low, and in combination with the short dwell time, this leads to a reduction in the SNR, which complicates the unambiguous detection of events. Although transient current blockages can be identified in the recording, their shape and short dwell time complicate the detection of individual translocation events, and some events may partially overlap with baseline fluctuations, or fall below the detection threshold. The observed behavior may also reflect the overall behavior of the electrolyte, including differences in ionic strength and ionic composition. It should be noted that even at higher 100 kHz acquisition bandwidth applied for Tris-based measurements, baseline noise was elevated, and residual noise contributions cannot be fully excluded from the smallest detected events. Notably, the shape of individual translocation events in the Tris-based system is qualitatively similar to that observed in LiCl/HEPES a relatively stable current level during translocation, and a return to baseline indicating that the detected events represent genuine translocation rather than noise artifacts. Tris-based buffer shows substantially shorter dwell time and lower blockage amplitude, which together reduce the signal-to-noise ratio and complicate reliable event discrimination. Figure 1 serves primarily as a qualitative demonstration of the nature of the signal in this buffer, while a more detailed quantitative analysis of these events is discussed in the following sections.

We note that a subset of events in the LiCl/HEPES data shows very long dwell times (up to ∼100 ms) and weak voltage dependence (visible in Supporting Information Figure S1), consistent with the subpopulation of surface interaction dominated events described by Skinner et al. for ssDNA in SiN nanopores.^40^ This behavior is characteristic of CDB fabricated pores and likely reflects transient trapping of the circular ssDNA at the nanopore surface. The faster events in the microseconds-to-millisecond range may represent events closer to free electrophoretic translocation. Figure 1 serves primarily as a qualitative demonstration of the signal character in each buffer.

Subsequent experiments compared the behavior of translocation events at different circular ssDNA concentrations and at different applied volatages in LiCl/HEPES buffer (Figure 2a–c). The number of detected translocation events increased with increasing (absolute) applied voltage across all concentrations (Table 1). Additionally, higher ssDNA concentrations also resulted in more frequent events as expected, with the most noticeable increase observed at −50 mV and −100 mV, while the effect was less evident at −80 mV. To provide a theoretical context for the observed trends, Supporting Information Figure S1 shows capture frequency as a function of ssDNA concentration and a median dwell time as a function of applied voltage for the LiCl/HEPES system. As shown in Supporting Information Figure S1a, capture frequency increases approximately linearly with concentration at all voltages, consistent with the diffusion limited capture model. The apparent nonlinearity visible in Table 1 at low concentration and low voltage reflects the small absolute number of events and the stochastic nature of individual 25 s recording windows. The high uncertainties at low concentrations and low voltages reflect the small absolute number of events, with the associated Poisson counting uncertainty. For example, at 3 nM and −50 mV in LiCl/HEPES (*N* = 5), the relative uncertainty in the capture frequency is approximately 45%. When the data are normalized to event frequency, the approximately linear concentration dependence becomes more apparent. We note that for these smallest data sets the fitted distributions in Figure 2d,e should be interpreted with caution. Supporting Information Figure S1b shows that the median dwell time decreases with increasing voltage, consistent with electrophoretic transport. A clear dependence of dwell time on applied voltage was observed, with increasing absolute voltage resulting in shorter translocation times. This trend is illustrated by the 5 nM data set, where median dwell time decreased from 9860 μs at −50 mV to 2580 μs at −80 mV, and further to 995 μs at −100 mV.

**Figure 2:**
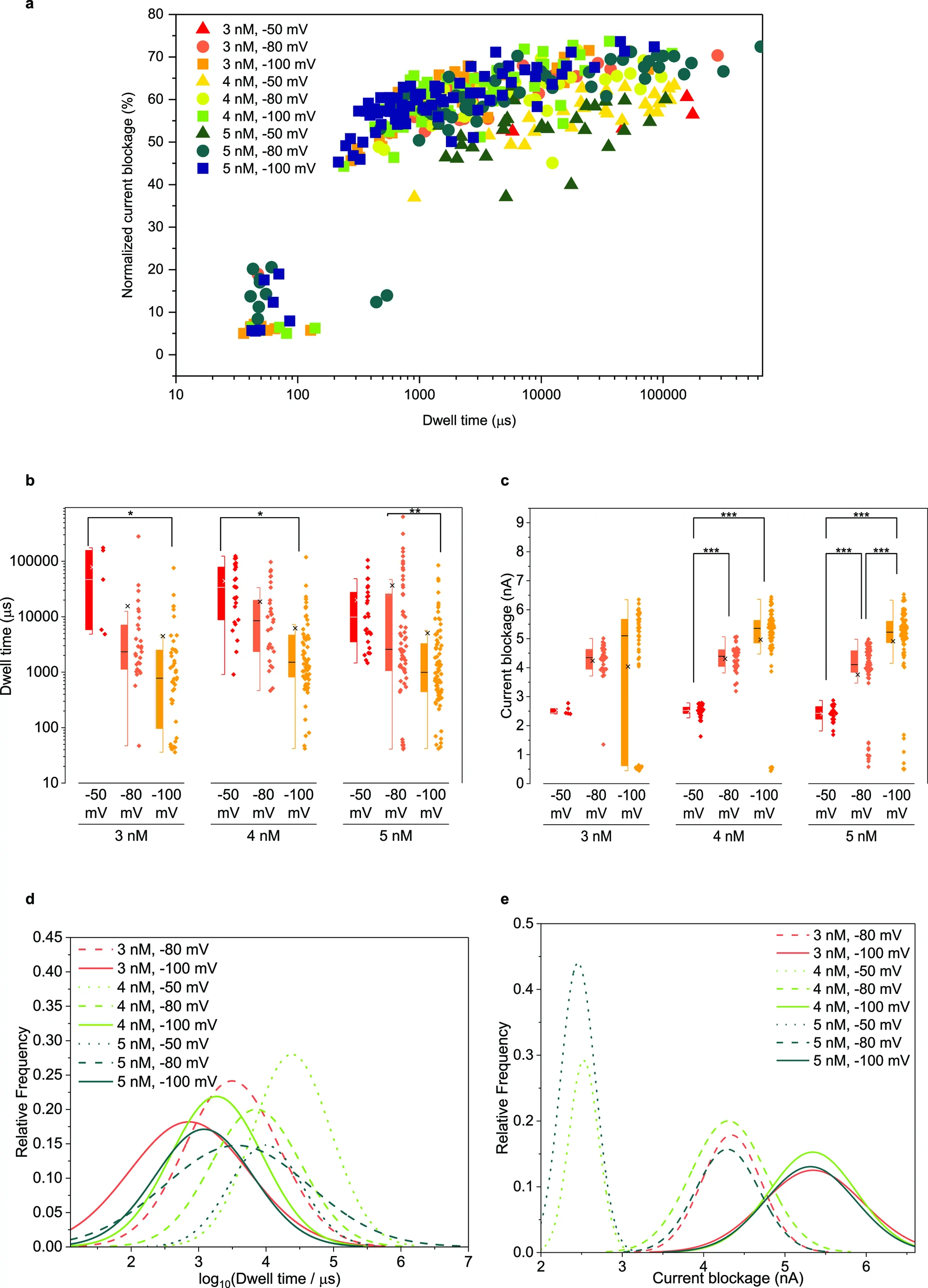
Dependence of circular ssDNA translocation on voltage and concentration in LiCl/HEPES buffer. (a) A scatter plot of individual translocation events at concentrations of 3, 4, and 5 nM in the voltage range –50 to –100 mV; (b–c) box plots of dwell times and current blockage amplitudes; (d–e) distributions of dwell times and current blockages showing voltage-dependent shift and signal variability. *p ≤ 0.05; **p ≤ 0.01; ***p ≤ 0.001, and the number of analyzed events in each measurement can be found from Table 1.

The voltage dependence of dwell time in LiCl/HEPES warrants a more detailed discussion in the context of the molecular properties of circular ssDNA. Kowalczyk et al. demonstrated that replacing K^+^ with Li^+^ cations slows DNA translocation by approximately 1 order of magnitude for linear dsDNA, which was attributed to stronger interactions of Li^+^ cations with the DNA phosphate backbone.^39^ However, since our analyte is a circular ssDNA rather than linear dsDNA, the translocation mechanism is expected to differ due to the presence of secondary structures.

Kowalczyk et al. further showed that long circular ssDNA molecules such as M13mp18 hybridize intramolecularly into compact blob-like structures with a diameter of approximately 100–150 nm. These structures were required to unravel before passing through the ∼7 nm nanopore, leading to an exponential dependence of dwell time on the absolute voltage $\left(\tau ∼e^{\left(-\left|V\right|/V_{0}\right)}\right)$ in their system, markedly different from the τ∼1/V relationship expected for friction dominated dsDNA transport.^41^ The circular ssDNA p7560 used in our study (7560 nt) carries the same nonrepetitive nucleotide sequence as M13mp18 (7249 nt) with a short 311 nt insert, and is thus structurally closely resembling M13mp18. Therefore, it is also assumed that p7560 forms a similarly compact secondary structure of ∼100–150 nm as M13mp18 through extensive intramolecular base pairing, analogous to the blob-like structures visualized by Kowalczyk et al. using AFM.^41^ In the LiCl/HEPES system, doubling the absolute potential (from −50 mV to −100 mV) leads to a factor of ∼10 reduction in the median dwell time at 5 nM (Supporting Information Figure S1b), and our data exhibit a power-law-like decrease in dwell time with increasing voltage rather than clear evidence of the exponential voltage dependence reported by Kowalczyk et al. for M13mp18 in ∼7 nm nanopore.^41^ This observation suggests that, in our system with a larger nanopore (∼16 nm), complete unraveling of compact ssDNA p7560 secondary structure may not represent the dominant rate-limiting step during translocation. The observed behavior is more consistent with electrophoretically driven transport, where interactions between Li^+^ cations and DNA, along with other effects related to the buffer composition, may contribute to the slower translocation observed in the system.^39,43^ Partial conformational rearrangements of the circular ssDNA molecule during translocation may additionally contribute to the observed dynamics.^43^ At a constant voltage, increasing ssDNA concentration led to slightly longer dwell times and increased variability, as indicated by broader interquartile range (IQR). Median current blockage amplitudes increased from 2.4 nA to 4.1 nA and 5.2 nA with increasing absolute voltage (from −50 mV to −100 mV).

Statistical analysis shows that the most pronounced differences in dwell time occurred between −50 mV and −100 mV across all concentrations, with the strongest effect observed at 5 nM. This confirms that voltage is the primary factor influencing translocation kinetics, while concentration primarily regulates variability and event rate. Figure 2a shows normalized current blockage (Δ*I*/*I*_0_, i.e., blockage normalized with respect to the baseline current). The majority of events cluster between 40–75%, with a much smaller fraction of events scattered down to ∼5%, likely corresponding to events involving partial translocations. The normalized blockage does not show a systematic increase with voltage, consistent with the interpretation that blockage amplitude is primarily determined by nanopore geometry and circular ssDNA conformation rather than by the applied electric field. The median/mean dwell times (Figure 2b,d) systematically decrease with increasing applied voltage, consistent with faster electrophoretic transport of molecules through the nanopore. Although the individual dwell time distributions span several orders of magnitude and partially overlap, the shift in median values clearly indicates a trend toward shorter translocation times at higher voltages. At a given voltage, median dwell times remain relatively similar across different concentrations, with concentration primarily affecting the event rate. This trend is further supported by Supporting Information Figure S2, where shorter characteristic dwell times are observed at −100 mV, while the distributions at −50 mV remain broader and shifted toward longer dwell times. The distribution of current blockage amplitudes (Figure 2c,e) shows a broad range of values, but remains similar across concentrations at a given voltage, indicating that the magnitude of the blockage amplitude is clearly dependent on the applied voltage and is primarily determined by the nanopore geometry and ssDNA’s conformation.

Next, an analysis of the translocation dynamics in Tris-based (0.5× TBE supplemented with 5.5 mM MgCl_2_, and 1 M KCl) electrolyte supplemented with K^+^ and Mg^2+^ ions (Figure 3a–c) at the same voltage range and circular ssDNA concentrations as above was carried out. Compared to LiCl/HEPES buffer, a substantially higher number of events was detected, increasing with both applied voltage and circular ssDNA concentration (Table 1), with the most counts obtained at −100 mV and 5 nM. The capture frequency increased with both applied voltage and circular ssDNA concentration, reaching a maximum value of ∼23 events/s at −100 mV and 5 nM (Supporting Information Figure S1a). Dwell times in the TBE system were markedly shorter than in LiCl/HEPES, remaining in the range of tens to hundreds of microseconds across all conditions. At 5 nM, the median dwell time decreased from 145 μs at −50 mV to 73 μs at −80 mV, and further to 72 μs at −100 mV, remaining well below the values observed in LiCl/HEPES at all voltages. The median dwell times in the TBE-based system were markedly shorter than in LiCl/HEPES, with the difference ranging from approximately 14-fold at −100 mV to nearly 70-fold at −50 mV (5 nM data set). As discussed above, the two buffer systems differ in cation type, ionic strength, and the presence of divalent Mg^2+^ cations.^38,45^ The observed differences therefore likely arise from the combined characteristics of the electrolyte environment rather than from a single isolated parameter. As discussed for LiCl/HEPES buffer, the work of Kowalczyk et al. provides a useful reference for interpreting the translocation behavior of circular ssDNA in 1 M KCl electrolytes.^41^ In their study, millisecond-scale dwell times were reported for M13mp18 translocation through a nanopore with a diameter of ∼7 nm.^41^ Since both studies use 1 M KCl as the dominant monovalent salt, the two systems are broadly comparable in ionic strength. The additional 5.5 mM MgCl_2_ present in the Tris-based buffer contributes only modestly to the total ionic strength relative to 1 M KCl but may influence ssDNA conformation through divalent cation interactions, as discussed above. Despite this similarity, the median dwell times in our TBE system (72–145 μs at 5 nM) are approximately 1 order of magnitude shorter than those observed in the LiCl/HEPES system, with larger differences occurring under specific experimental condition. We suggest this difference is primarily associated with the substantially larger diameter of the nanopore used in our study (∼16 nm compared to ∼7 nm in the work by Kowalczyk et al.^41^), which corresponds to more than double the diameter and approximately to five times the cross-sectional nanopore area. The larger pore diameter substantially reduces geometric confinement which may additionally facilitate faster molecular passage through reduced confinement effects.^40^ The presence of Mg^2+^ in the TBE system likely stabilizes the compact secondary structure of circular ssDNA by shielding the charge of the phosphate backbone. However, this contribution cannot be distinguished from the other simultaneous differences between the two electrolyte systems (cation composition, ionic strength, salt concentration).^45^ The larger nanopore diameter used in the present study substantially reduces geometric confinement relative to the nanopore used by Kowalczyk et al., which likely contributes to the observed faster translocation dynamics.^41^ The larger pore diameter is also expected to reduce confinement and potentially decrease DNA-surface interactions. Overall, these observations show that the translocation dynamics of long circular ssDNA depend on a combination of buffer composition, pore geometry, ssDNA-surface interactions, and the secondary structure of the molecule, with the relative contribution of each factor varying according to the specific experimental conditions.

**Figure 3:**
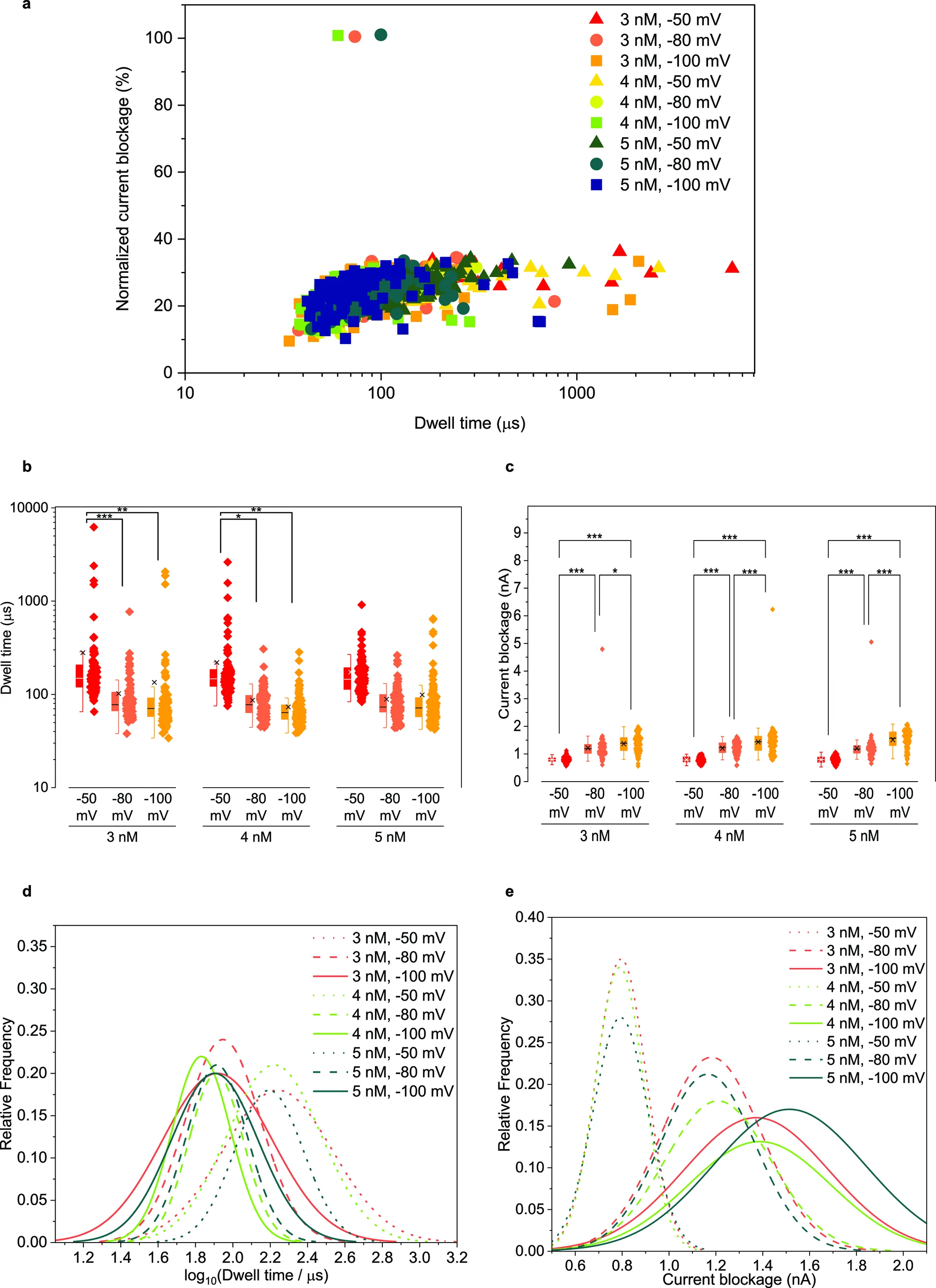
Dependence of ssDNA translocation on applied voltage and DNA concentration in TBE buffer supplemented with Mg^2+^ and K^+^ cations. (a) A scatter plot of individual translocation events at concentrations of 3, 4, and 5 nM in the voltage range −50 to −100 mV; (b–c) Box plots of dwell times and current blockage amplitudes; (d–e) distributions of dwell times and current blockages showing voltage-dependent shift and signal variability. *p ≤ 0.05; **p ≤ 0.01; ***p ≤ 0.001, and the number of analyzed events in each measurement can be found from Table 1.

The overall voltage dependence of dwell time was weaker than in LiCl/HEPES, with partial overlap between −80 mV and −100 mV distributions (Supporting Information Figure S1b). At a given voltage, higher concentrations led to broader IQR and longer whiskers, indicating increased variability rather than a systematic shift in median dwell time, suggesting that higher concentrations increase the diversity of individual translocation events, likely due to varying DNA-pore interactions rather than a systematic slowing of transport. Figure 3a shows the normalized current blockage (Δ*I*/*I*_0_) for the Tris-based system. The majority of events form a cluster between approximately 15–35%, reflecting the reduced blockage amplitude and higher charge screening in this buffer. A smaller number of events extend to higher normalized values, but no systematic voltage dependence of the normalized blockage is observed, consistent with the interpretation that blockage amplitude is governed primarily by nanopore geometry and circular ssDNA conformation. Overall, median current blockage amplitudes were lower and fell within a narrower range from 0.8 nA to 1.2 nA and 1.6 nA (Figure 3c,e), consistent with the altered ion transport and higher charge screening in the buffer. The current blockage increased clearly with applied voltage across all concentrations, with clear differences observed between −50 mV and −100 mV (***p ≤ 0.001), between −50 mV and −80 mV (***p ≤ 0.001), and between −80 mV and −100 mV (***p ≤ 0.001). In contrast, differences between concentration at a given voltage were not statistically relevant, confirming that current blockage in the TBE-system is determined primarily by the applied voltage, not by circular ssDNA concentration. The reduced amplitude is consistent with the overall properties of the Tris-based buffer, including its ionic strength and ionic composition. However, the contribution of individual components cannot be distinguished in this study. The median/mean dwell times (Figure 3b,d) decreased systematically with increasing applied voltage. The overall voltage dependence of the median dwell time was weaker than in LiCl/HEPES, as reflected by the smaller relative change between −80 mV and −100 mV, with partial overlap between the corresponding dwell time distributions. At a given voltage, median dwell times remained relatively similar across different concentrations, with concentration primarily affecting the event rate rather than the characteristic dwell time. This trend is further supported by the Supporting Information Figure S3, where shorter median dwell times are observed at −100 mV, while the distributions at −50 mV are broader and shifted toward longer dwell times, consistent with the voltage dependent behavior observed in LiCl/HEPES, though confined to a substantially narrower time window of tens to hundreds of microseconds.

The current blockage distributions (Figure 3e) are likewise narrow and largely overlapping across concentrations at each voltage, consistent with the interpretation that blockage amplitude in the TBE system is determined primarily by nanopore geometry and ssDNA conformation rather than by concentration. Overall, translocation in the TBE-based buffer occurs within a narrow time window, reducing the influence of concentration and limiting the resolution of individual events compared to LiCl/HEPES buffer.

## 4 Conclusion

This study clearly demonstrates that increasing the applied bias voltage alone is not sufficient to ensure high-quality nanopore measurements if the buffer is not appropriately selected. While a high negative voltage accelerates translocation and increases current blockage, the detectability and interpretability of events are largely determined by the overall electrolyte environment. In LiCl/HEPES, circular ssDNA translocation events are longer and exhibit higher amplitudes and thus are easily distinguishable from noise at a standard acquisition bandwidth of 50 kHz. In contrast, the Tris-borate system supplemented with K^+^ and Mg^2+^ ions results in very fast translocation, which together reduce the SNR, necessitating a higher acquisition bandwidth of 100 kHz and stricter detection threshold to detect individual events reliably. We emphasize that the two buffer systems differ in multiple parameters simultaneously (cation type, salt concentration, ionic strength, and presence of Mg^2+^ ions), and therefore the observed differences characterize the two complete ionic environments rather than an effect of any single variable. Controlled experiments are isolating individual variables are planned for future work. The capture frequency scales are approximately linearly with circular ssDNA concentration, consistent with the diffusion-limited capture model, and dwell time decreases with increasing absolute voltage following a power-law relationship consistent with electrophoretic transport. A subpopulation of long events in LiCl/HEPES with weak voltage dependence is consistent with DNA–surface interactions in CDB-fabricated SiN nanopores.^40^ Furthermore, compared with the exponential voltage dependence reported by Kowalczyk et al. for M13mp18 translocating through a ∼7 nm nanopore,^41^ the power-law voltage dependence of dwell time observed in LiCl/HEPES suggests that given the larger pore diameter used in this study (∼16 nm), complete unraveling of the compact secondary structure of circular ssDNA is unlikely to represent the dominant rate-limiting step in translocation. The observed voltage dependence is instead more consistent with electrophoretically driven transport, where interactions between Li^+^ cations and DNA^39^ may contribute to the observed slowing, along with other effects related to the electrolyte composition and with a possible additional contribution from partial conformational rearrangements of the circular ssDNA molecule during translocation.^43^ In contrast, the Tris-based system showed markedly shorter dwell times, predominantly in the range of tens to hundreds of microseconds, together with lower current blockage amplitudes. In this case, the combination of a different ionic environment and the larger nanopore diameter with reduced geometric confinement result in fast molecular translocation.

DNA concentration primarily affects the event rate, whereas the characteristics of individual events are mainly governed by the applied voltage and ionic environment. For reliable single-molecule detection, it is therefore essential to select conditions that prolong dwell times and increase the blockage amplitude. Overall, these findings provide practical guideline for designing reliable SSNP experiments for single-molecule detection of long circular ssDNA and are transferable to other related applications, including future integration of DNA origami structures into solid-state nanopore. In our system, this is achieved using LiCl/HEPES, which allows reliable detection and characterization of translocation events of long circular ssDNA at a standard 50 kHz acquisition setting, whereas when using Tris-borate system supplemented with K^+^ and Mg^2+^ ions, it is necessary to reduce the voltage and/or increase the acquisition bandwidth and apply stricter detection thresholds to capture very short and low amplitude events.

## Supplementary Material



## Notes

Capture frequency as a function of circular ssDNA concentration; median dwell time as a function of applied voltage; histograms of dwell times and current blockages for circular ssDNA translocation in LiCl/HEPES and Tris-based buffers (PDF)
