# Supplementary material for: Effect of Electrolyte, Applied Voltage, and DNA Concentration on the Translocation Dynamics of a Single-Stranded DNA through a Solid-State Nanopore
Source: ACS Omega. 2026 Aug 13;11(33):49706–16. doi: 10.1021/acsomega.6c03954 (PMC13520439; doi:10.1021/acsomega.6c03954)
Supplement: Supplementary file 1 [file ao6c03954_si_001.pdf]

Supporting Information

Effect of Electrolyte, Applied Voltage, and DNA Concentration on the Translocation Dynamics of a Single-Stranded DNA through a Solid-State Nanopore

Agnes Zerolová [a], Shubham Mondal [a], Petteri Piskunen [a], Mauri A. Kostiainen [a,] and Veikko Linko [a,b,*]*

[a]Department of Bioproducts and Biosystems, Aalto University, 00076 Aalto, Finland

[b]Institute of Technology, University of Tartu, 50411 Tartu, Estonia

*Corresponding author e-mails: mauri.kostiainen@aalto.fi; veikko.pentti.linko@ut.ee

Table of Contents

Figure S1. Capture frequency and dwell time scaling for circular ssDNA in 3.6 M LiCl, 10 mM HEPES buffer and 0.5×TBE, 5.5 mM $MgCl_2$, and 1 M KCl buffer.

Figure S2. Histograms of dwell times and current blockages for circular ssDNA translocation in 3.6 M LiCl, 10 mM HEPES buffer at various applied voltages (−100 mV (a–b), −80 mV (c–d), and −50 mV (e–f)).

Figure S3. Histograms of dwell times and current blockages for circular ssDNA translocation in 0.5× TBE, 5.5 mM $MgCl_2$, 1 M KCl buffer at various applied voltages (−100 mV (a–b), −80 mV (c–d), and −50 mV (e–f)).

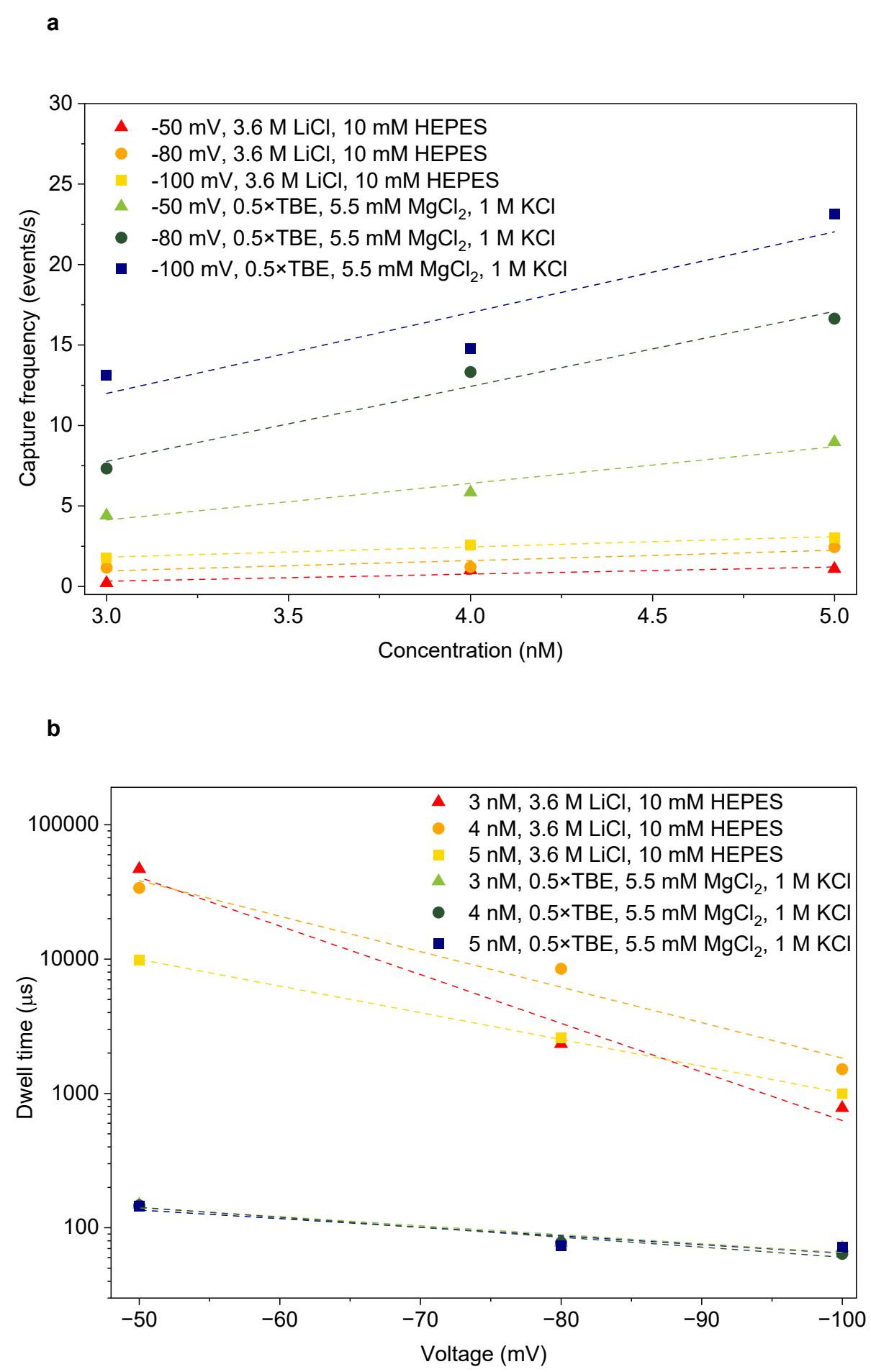

Figure S1. Capture frequency and dwell time scaling for circular ssDNA in 3.6 M LiCl, 10 mM HEPES buffer and 0.5×TBE, 5.5 mM $MgCl_2$, and 1 M KCl buffer. a) Capture frequency (events/s) as a function of circular ssDNA concentration (3–5 nM) at three applied voltages (−50, −80 and −100 mV). The dashed lines are proportional fits passing through the origin. The error bars represent the Poisson counting uncertainty. b) Median dwell time as a function of applied voltage for circular ssDNA concentrations (3–5 nM), showing a power-law-like decrease consistent with electrophoretic transport. The error bars represent the interquartile range of the distribution of dwell times.

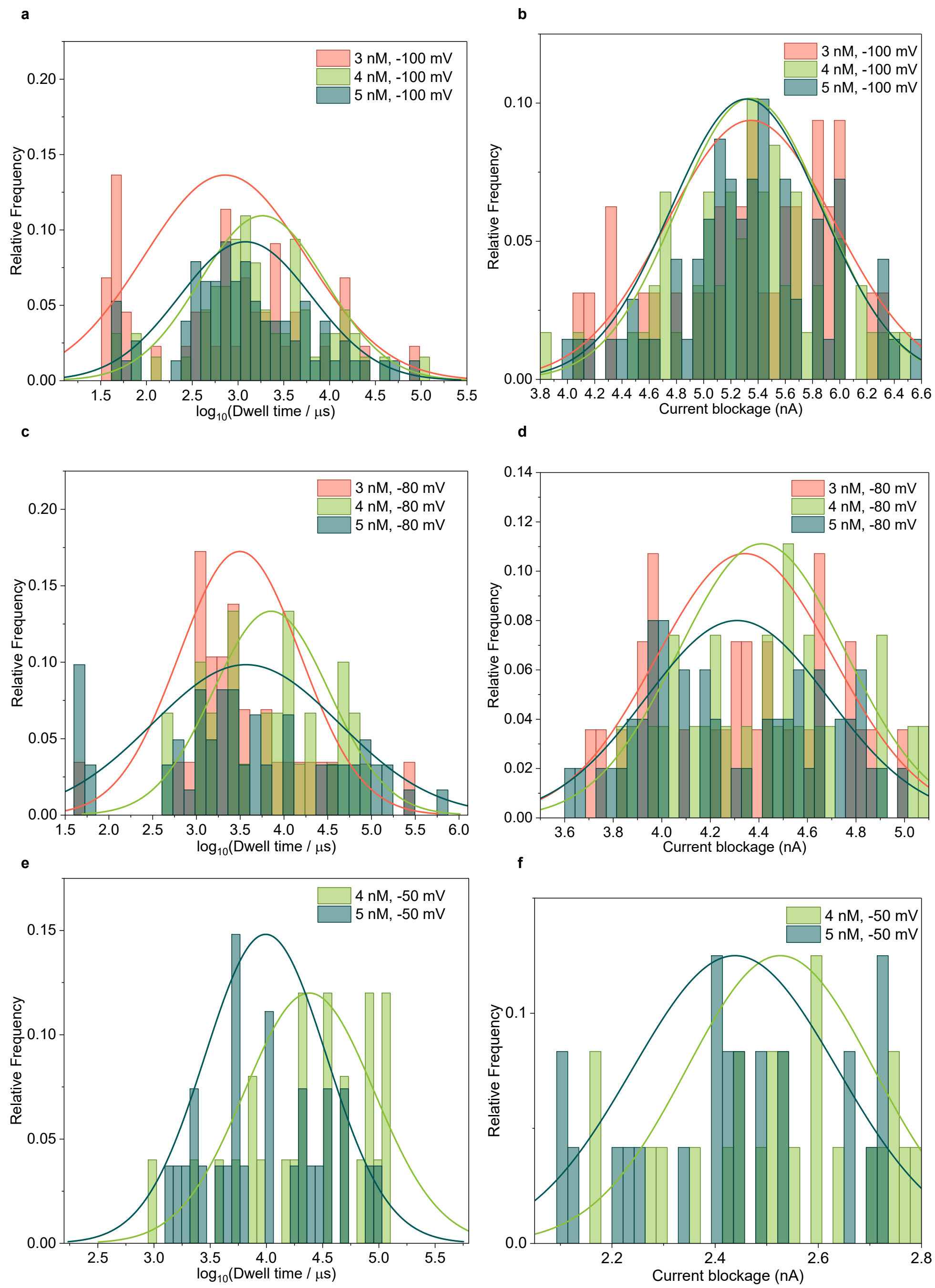

Figure S2. Histograms of dwell times (left) and current blockages (right) for circular ssDNA translocation in 3.6 M LiCl, 10 mM HEPES buffer at various applied voltages (−100 mV (a–b), −80 mV (c–d), and −50 mV (e–f)).

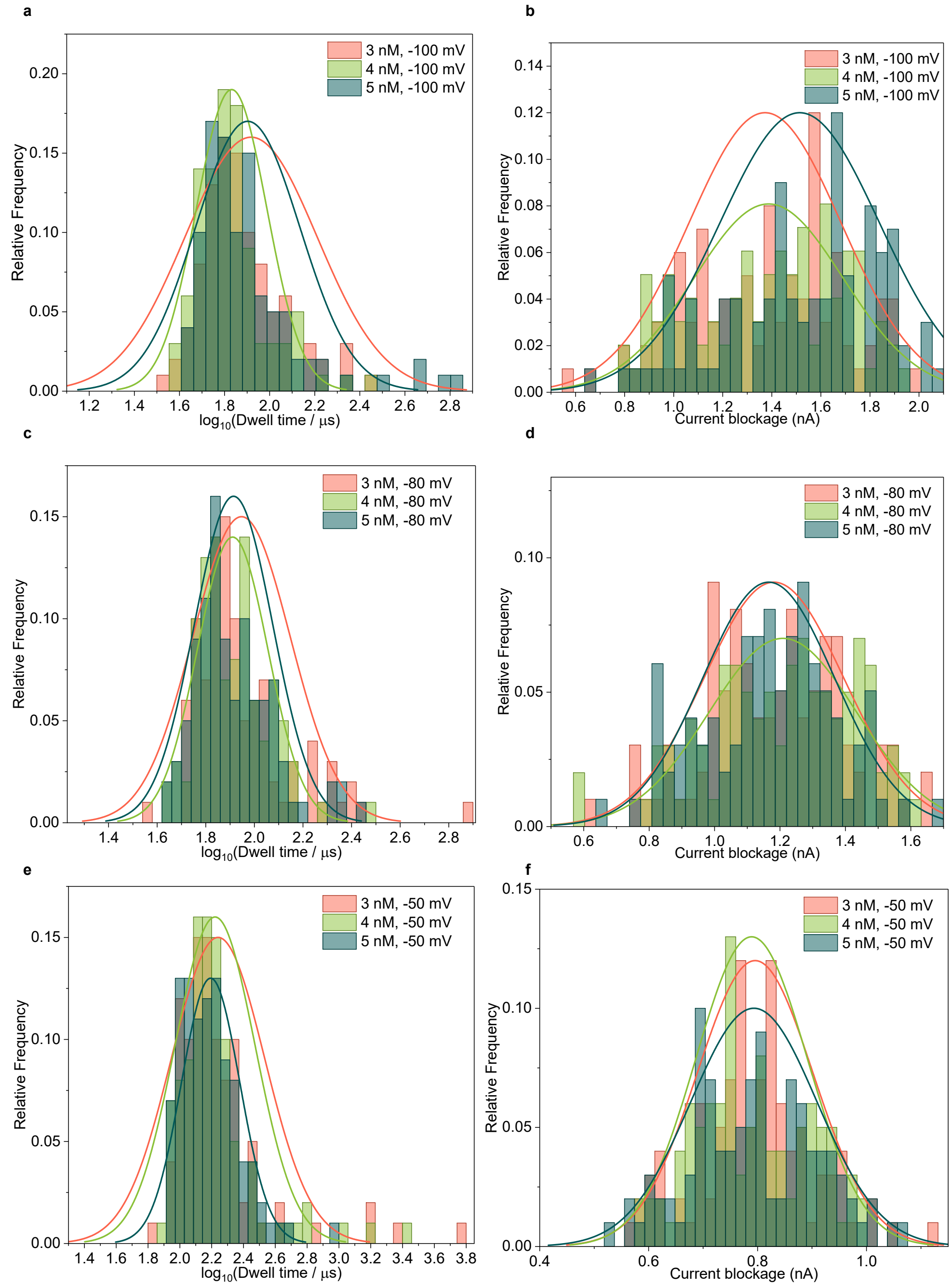

Figure S3. Histograms of dwell times (left) and current blockages (right) for circular ssDNA translocation in 0.5× TBE, 5.5 mM $MgCl_2$, 1 M KCl buffer at various applied voltages (−100 mV (a–b), −80 mV (c–d), and −50 mV (e–f)).
